# A new polymorph of 2,6-dimeth­oxy­benzoic acid

**DOI:** 10.1107/S1600536811049075

**Published:** 2011-11-23

**Authors:** Gustavo Portalone

**Affiliations:** a Chemistry Department, "Sapienza" University of Rome, P.le A. Moro, 5, I-00185 Rome, Italy

## Abstract

A new crystalline form of 2,6-dimeth­oxy­benzoic acid, C_9_H_10_O_4_, crystallizing in a tetra­gonal unit cell has been identified during screening for co-crystals. The asymmetric unit comprises a non-planar independent mol­ecule with a synplanar conformation of the carb­oxy group. The sterically bulky *o*-meth­oxy substituents force the carb­oxy group to be twisted away from the plane of the benzene ring by 65.72 (15)°. The carb­oxy group is disordered over two sites about the C—C bond [as indicated by the almost equal C—O distances of 1.254 (3) and 1.250 (3) Å], the occupancies of the disordered carboxym H atoms being 0.53 (5) and 0.47 (5). In the known ortho­rhom­bic form reported by Swaminathan *et al.* [*Acta Cryst.* (1976), B**32**, 1897–1900], due to the anti­planar conformation adopted by the OH group, the mol­ecular components are associated in the crystal in chains stabilized by linear O—H⋯O hydrogen bonds. However, in the new tetra­gonal polymorph, mol­ecules form dimeric units *via* pairs of O—H⋯O hydrogen bonds between the carb­oxy groups.

## Related literature

For the ortho­rhom­bic polymorph of 2,6-dimeth­oxy­benzoic acid, see: Swaminathan *et al.* (1976 ▶); Bryan & White (1982 ▶); Portalone (2009 ▶). For mol­ecular packing modes of carb­oxy­lic acids, see: Leiserowitz (1976 ▶); Kanters *et al.* (1991 ▶); Moorthy *et al.* (2002 ▶). For analysis of benzene ring deformations induced by substitution, see: Schultz *et al.* (1993 ▶); Portalone *et al.* (1998 ▶); For computation of ring patterns formed by hydrogen bonds in crystal structures, see: Etter *et al.* (1990 ▶); Bernstein *et al.* (1995 ▶); Motherwell *et al.* (1999 ▶).
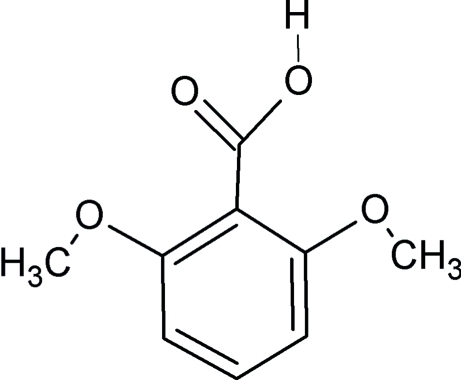


## Experimental

### 

#### Crystal data

C_9_H_10_O_4_
*M* *_r_* = 182.17
Tetragonal, 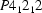

*a* = 8.1423 (3) Å
*c* = 27.6814 (18) Å
*V* = 1835.20 (15) Å^3^
*Z* = 8
Mo *K*α radiation
μ = 0.11 mm^−1^
*T* = 298 K
0.30 × 0.25 × 0.21 mm

#### Data collection

Oxford Diffraction Xcalibur S CCD diffractometer
Absorption correction: multi-scan (*CrysAlis RED*; Oxford Diffraction, 2006 ▶). *T*_min_ = 0.878, *T*_max_ = 0.999
11616 measured reflections
1653 independent reflections
1332 reflections with *I* > 2σ(*I*)
*R*_int_ = 0.042

#### Refinement

*R*[*F* ^2^ > 2σ(*F* ^2^)] = 0.062
*wR*(*F* ^2^) = 0.147
*S* = 1.19
1653 reflections
133 parameters
H atoms treated by a mixture of independent and constrained refinement
Δρ_max_ = 0.18 e Å^−3^
Δρ_min_ = −0.17 e Å^−3^

### 

Data collection: *CrysAlis CCD* (Oxford Diffraction, 2006 ▶); cell refinement: *CrysAlis RED* (Oxford Diffraction, 2006 ▶); data reduction: *CrysAlis RED*; program(s) used to solve structure: *SIR97* (Altomare *et al.*, 1999 ▶); program(s) used to refine structure: *SHELXL97* (Sheldrick, 2008 ▶); molecular graphics: *ORTEP-3 for Windows* (Farrugia, 1997 ▶); software used to prepare material for publication: *WinGX* (Farrugia, 1999 ▶).

## Supplementary Material

Crystal structure: contains datablock(s) I, global. DOI: 10.1107/S1600536811049075/xu5390sup1.cif

Structure factors: contains datablock(s) I. DOI: 10.1107/S1600536811049075/xu5390Isup2.hkl

Supplementary material file. DOI: 10.1107/S1600536811049075/xu5390Isup3.cml

Additional supplementary materials:  crystallographic information; 3D view; checkCIF report

## Figures and Tables

**Table 1:** Hydrogen-bond geometry (Å, °)

*D*—H⋯*A*	*D*—H	H⋯*A*	*D*⋯*A*	*D*—H⋯*A*
O1—H1⋯O1^i^	0.77 (4)	1.87 (4)	2.632 (4)	168 (5)
O2—H2⋯O2^i^	0.79 (5)	1.83 (5)	2.618 (4)	173 (5)

Symmetry code: (i) 
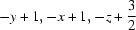
.

**Fig. 1.**
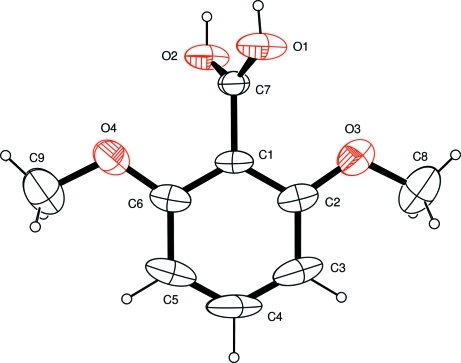
The molecular structure of (I), showing the atom-labelling scheme. Displacements ellipsoids are at the 50% probability level.

**Fig. 2.**
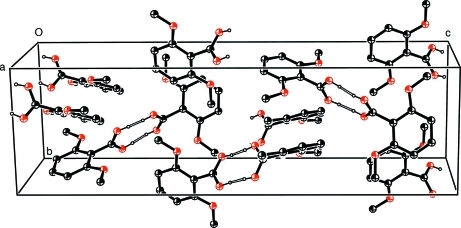
Crystal packing diagram for (I) viewed approximately down a. All atoms are shown as small spheres of arbitrary radii. For the sake of clarity, H atoms not involved in hydrogen bonding have been omitted. Hydrogen bonding is indicated by dashed lines.

**Fig. 3.**
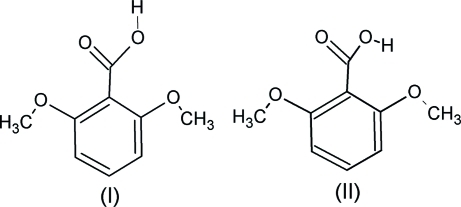
A scheme showing antiplanar and synplanar conformations of the carboxy group.

## supplementary crystallographic information

### Comment

In this paper it is reported the crystal structure of a new polymorph, (I), of 2,6-dimethoxybenzoic acid, produced unexpectedly during an attempt to synthesize cocrystals of boronic acid with of 2,6-dimethoxybenzoic acid. The known form, (II) (Fig. 3), of of 2,6-dimethoxybenzoic acid is orthorhombic in the space group *P*2_1_2_1_2_1_ and crystallizes with one molecule in the asymmetric unit (Swaminathan *et al.*, 1976; Bryan & White, 1982; Portalone, 2009). In (II), due to the antiplanar conformation adopted by the OH group, the molecular components are associated in the crystal in chains stabilized by linear O—H···O hydrogen bonds.

The title new polymorph (I) is tetragonal in the space group P4_1_2_1_2. The asymmetric unit of (I) comprises a non-planar independent molecule, as the *o*-methoxy substituents force the carboxy group to be twisted away from the plane of the phenyl ring by 65.72 (15)° (Fig. 1). The carboxy group, which adopts a synplanar conformation, is almost completely disordered, as indicated by the equal C—O distances, 1.254 (3) and 1.250 (3) Å, the C—C—O angles, 118.9 (2) and 117.8 (2)°, and by the presence of disordered H atoms with occupancy factors of 0.53 (5) and 0.47 (5) in the O···O intermolecular hydrogen bond. The pattern of bond lengths and bond angles of the phenyl ring is consistent with that reported in the structure determination of (II), and a comparison of the present results with those obtained for similar benzene derivatives in the gas phase (Schultz *et al.*, 1993; Portalone *et al.*, 1998) shows no appreciable effects of the crystal environment on the ring deformation induced by substituents. Analysis of the crystal packing of (I), (Fig. 2), shows that the molecular components form the conventional dimeric units observed in benzoic acids (Leiserowitz, 1976; Kanters *et al.*, 1991; Moorthy *et al.*, 2002). Indeed, the structure is stabilized by usual intermolecular C^2^_2_(8) O—H···O interactions (Etter *et al.*, 1990; Bernstein *et al.*, 1995; Motherwell *et al.*, 1999) (Table 1) which link the molecules into dimers through the disordered carboxy moieties [symmetry code: (i) -*y* + 1, -*x* + 1, -*z* + 3/2].

### Experimental

Polymorph (I) was formed during cocrystallization in a 1:1 molar ratio of 2,6-dimethoxybenzoic acid (1 mmol, Sigma Aldrich at 99% purity) and phenylboronic acid (1 mmol, Sigma Aldrich at 97% purity). The two components were dissolved in water (10 ml) and gently heated under reflux for 3 h. After cooling the solution to an ambient temperature, only one crystal suitable for single-crystal X-ray diffraction was grown by slow evaporation of the solvent after two weeks. Unfortunately, any attempts to produce more crystals of polymorph (I) by repeating the crystallization conditions were unsuccessful. Crystallization of 2,6-dimethoxybenzoic acid carried out under a wide range of different sets of conditions (different solvents, different molar ratio, different cosolute molecules) led systematically to the orthorhombic polymorph.

### Refinement

All H atoms were identified in difference Fourier maps, but for refinement all C-bound H atoms were placed in calculated positions, with C—H = 0.97 Å (phenyl) and 0.97–0.98 Å (methyl), and refined as riding on their carrier atoms. The *U*_iso_ values were kept equal to 1.2*U*_eq_(C, phenyl). and to 1.5*U*_eq_(C, methyl). The remaining two half H atoms of the carboxy group were freely refined and their occupancy factors constrained to sum to unity. In the absence of significant anomalous scattering in this light-atom study, Friedel pairs were merged.

### Crystal data

**Table:**

C_9_H_10_O_4_	*D*_x_ = 1.319 Mg m^−^^3^
*M**_r_* = 182.17	Mo *K*α radiation, λ = 0.71069 Å
Tetragonal, *P*4_1_2_1_2	Cell parameters from 4278 reflections
Hall symbol: P 4abw 2nw	θ = 2.9–32.3°
*a* = 8.1423 (3) Å	µ = 0.11 mm^−^^1^
*c* = 27.6814 (18) Å	*T* = 298 K
*V* = 1835.20 (15) Å^3^	Tablets, colourless
*Z* = 8	0.30 × 0.25 × 0.21 mm
*F*(000) = 768	

### Data collection

**Table:**

Oxford Diffraction Xcalibur S CCD diffractometer	1653 independent reflections
Radiation source: Enhance (Mo) X-ray source	1332 reflections with *I* > 2σ(*I*)
graphite	*R*_int_ = 0.042
Detector resolution: 16.0696 pixels mm^-1^	θ_max_ = 30.0°, θ_min_ = 2.9°
ω and φ scans	*h* = −11→10
Absorption correction: multi-scan (*CrysAlis RED*; Oxford Diffraction, 2006).	*k* = −8→11
*T*_min_ = 0.878, *T*_max_ = 0.999	*l* = −38→38
11616 measured reflections	

### Refinement

**Table:**

Refinement on *F*^2^	Secondary atom site location: difference Fourier map
Least-squares matrix: full	Hydrogen site location: inferred from neighbouring sites
*R*[*F*^2^ > 2σ(*F*^2^)] = 0.062	H atoms treated by a mixture of independent and constrained refinement
*wR*(*F*^2^) = 0.147	*w* = 1/[σ^2^(*F*_o_^2^) + (0.0659*P*)^2^ + 0.2634*P*] where *P* = (*F*_o_^2^ + 2*F*_c_^2^)/3
*S* = 1.19	(Δ/σ)_max_ < 0.001
1653 reflections	Δρ_max_ = 0.18 e Å^−^^3^
133 parameters	Δρ_min_ = −0.17 e Å^−^^3^
0 restraints	Extinction correction: *SHELXL97* (Sheldrick, 2008), Fc^*^=kFc[1+0.001xFc^2^λ^3^/sin(2θ)]^-1/4^
Primary atom site location: structure-invariant direct methods	Extinction coefficient: 0.017 (3)

### Special details

**Table:**

Geometry. All s.u.'s (except the s.u. in the dihedral angle between two l.s. planes) are estimated using the full covariance matrix. The cell s.u.'s are taken into account individually in the estimation of s.u.'s in distances, angles and torsion angles; correlations between s.u.'s in cell parameters are only used when they are defined by crystal symmetry. An approximate (isotropic) treatment of cell s.u.'s is used for estimating s.u.'s involving l.s. planes.
Refinement. Refinement of *F*^2^ against ALL reflections. The weighted *R*-factor *wR* and goodness of fit *S* are based on *F*^2^, conventional *R*-factors *R* are based on *F*, with *F* set to zero for negative *F*^2^. The threshold expression of *F*^2^ > 2σ(*F*^2^) is used only for calculating *R*-factors(gt) *etc*. and is not relevant to the choice of reflections for refinement. *R*-factors based on *F*^2^ are statistically about twice as large as those based on *F*, and *R*- factors based on ALL data will be even larger.

### Fractional atomic coordinates and isotropic or equivalent isotropic displacement parameters (Å^2^)

**Table:**

	*x*	*y*	*z*	*U*_iso_*/*U*_eq_	Occ. (<1)
O1	0.7090 (3)	0.1661 (3)	0.71019 (8)	0.0558 (6)	
H1	0.733 (5)	0.209 (5)	0.7340 (15)	0.023 (14)*	0.53 (5)
O2	0.5271 (3)	0.3665 (2)	0.70822 (7)	0.0508 (6)	
H2	0.566 (7)	0.393 (7)	0.7333 (17)	0.031 (18)*	0.47 (5)
O3	0.7742 (3)	0.2435 (3)	0.60828 (6)	0.0652 (7)	
O4	0.2838 (3)	0.1176 (3)	0.68526 (7)	0.0618 (6)	
C1	0.5250 (3)	0.1775 (3)	0.64409 (7)	0.0362 (6)	
C2	0.6221 (4)	0.1801 (3)	0.60276 (8)	0.0456 (7)	
C3	0.5579 (5)	0.1225 (4)	0.55922 (9)	0.0644 (9)	
H3	0.6230	0.1245	0.5299	0.077*	
C4	0.3993 (6)	0.0628 (5)	0.55889 (11)	0.0749 (11)	
H4	0.3547	0.0220	0.5287	0.090*	
C5	0.3025 (5)	0.0583 (4)	0.59895 (12)	0.0653 (9)	
H5	0.1917	0.0147	0.5972	0.078*	
C6	0.3652 (4)	0.1176 (3)	0.64256 (9)	0.0447 (7)	
C7	0.5920 (3)	0.2410 (3)	0.69057 (7)	0.0329 (5)	
C8	0.8729 (5)	0.2678 (4)	0.56701 (12)	0.0722 (11)	
H8A	0.889 (3)	0.164 (2)	0.5507 (7)	0.108*	
H8B	0.978 (3)	0.312 (3)	0.5767 (3)	0.108*	
H8C	0.819 (2)	0.344 (3)	0.5453 (7)	0.108*	
C9	0.1147 (5)	0.0710 (6)	0.68556 (15)	0.0917 (14)	
H9A	0.0518 (15)	0.147 (3)	0.6655 (11)	0.138*	
H9B	0.0730 (16)	0.074 (4)	0.7186 (7)	0.138*	
H9C	0.1036 (7)	−0.040 (3)	0.6728 (11)	0.138*	

### Atomic displacement parameters (Å^2^)

**Table:**

	*U*^11^	*U*^22^	*U*^33^	*U*^12^	*U*^13^	*U*^23^
O1	0.0679 (14)	0.0562 (13)	0.0432 (9)	0.0162 (11)	−0.0267 (10)	−0.0181 (9)
O2	0.0616 (13)	0.0497 (12)	0.0412 (9)	0.0118 (10)	−0.0217 (10)	−0.0159 (9)
O3	0.0665 (15)	0.0862 (17)	0.0428 (10)	−0.0139 (13)	0.0104 (10)	−0.0077 (11)
O4	0.0504 (13)	0.0836 (16)	0.0513 (10)	−0.0242 (12)	−0.0085 (10)	0.0041 (11)
C1	0.0506 (15)	0.0330 (12)	0.0251 (9)	−0.0008 (11)	−0.0133 (10)	−0.0031 (9)
C2	0.0618 (18)	0.0448 (15)	0.0302 (10)	0.0038 (14)	−0.0069 (12)	−0.0045 (11)
C3	0.094 (3)	0.069 (2)	0.0306 (12)	0.014 (2)	−0.0103 (15)	−0.0175 (14)
C4	0.095 (3)	0.083 (2)	0.0461 (16)	0.007 (2)	−0.0347 (18)	−0.0298 (17)
C5	0.068 (2)	0.064 (2)	0.0636 (17)	−0.0076 (16)	−0.0356 (17)	−0.0177 (17)
C6	0.0530 (17)	0.0412 (14)	0.0400 (12)	−0.0028 (13)	−0.0173 (12)	−0.0035 (11)
C7	0.0373 (13)	0.0370 (13)	0.0242 (8)	−0.0047 (10)	−0.0050 (9)	−0.0023 (9)
C8	0.095 (3)	0.057 (2)	0.0641 (18)	−0.0006 (19)	0.0319 (19)	−0.0022 (17)
C9	0.063 (3)	0.123 (4)	0.089 (3)	−0.034 (2)	−0.007 (2)	0.003 (3)

### Geometric parameters (Å, °)

**Table:**

O1—C7	1.254 (3)	C3—C4	1.380 (6)
O1—H1	0.77 (4)	C3—H3	0.9700
O2—C7	1.250 (3)	C4—C5	1.361 (5)
O2—H2	0.79 (5)	C4—H4	0.9700
O3—C2	1.350 (4)	C5—C6	1.397 (4)
O3—C8	1.411 (4)	C5—H5	0.9700
O4—C6	1.355 (4)	C8—H8A	0.9684
O4—C9	1.428 (5)	C8—H8B	0.9684
C1—C2	1.391 (4)	C8—H8C	0.9684
C1—C6	1.391 (4)	C9—H9A	0.9766
C1—C7	1.490 (3)	C9—H9B	0.9766
C2—C3	1.395 (4)	C9—H9C	0.9766
			
C7—O1—H1	110 (3)	O4—C6—C1	115.5 (2)
C7—O2—H2	113 (4)	O4—C6—C5	125.1 (3)
C2—O3—C8	119.0 (2)	C1—C6—C5	119.3 (3)
C6—O4—C9	118.5 (3)	O2—C7—O1	123.3 (2)
C2—C1—C6	120.8 (2)	O2—C7—C1	117.8 (2)
C2—C1—C7	119.8 (2)	O1—C7—C1	118.9 (2)
C6—C1—C7	119.4 (2)	O3—C8—H8A	109.5
O3—C2—C1	115.7 (2)	O3—C8—H8B	109.5
O3—C2—C3	124.8 (3)	H8A—C8—H8B	109.5
C1—C2—C3	119.5 (3)	O3—C8—H8C	109.5
C4—C3—C2	118.4 (3)	H8A—C8—H8C	109.5
C4—C3—H3	120.8	H8B—C8—H8C	109.5
C2—C3—H3	120.8	O4—C9—H9A	109.5
C5—C4—C3	123.1 (3)	O4—C9—H9B	109.5
C5—C4—H4	118.5	H9A—C9—H9B	109.5
C3—C4—H4	118.5	O4—C9—H9C	109.5
C4—C5—C6	118.9 (3)	H9A—C9—H9C	109.5
C4—C5—H5	120.6	H9B—C9—H9C	109.5
C6—C5—H5	120.6		
			
C8—O3—C2—C1	−172.8 (3)	C9—O4—C6—C5	−7.4 (5)
C8—O3—C2—C3	5.7 (5)	C2—C1—C6—O4	178.5 (3)
C6—C1—C2—O3	178.9 (2)	C7—C1—C6—O4	−2.0 (4)
C7—C1—C2—O3	−0.6 (4)	C2—C1—C6—C5	0.4 (4)
C6—C1—C2—C3	0.3 (4)	C7—C1—C6—C5	179.9 (3)
C7—C1—C2—C3	−179.2 (3)	C4—C5—C6—O4	−178.6 (3)
O3—C2—C3—C4	−179.2 (3)	C4—C5—C6—C1	−0.8 (5)
C1—C2—C3—C4	−0.8 (5)	C2—C1—C7—O2	114.4 (3)
C2—C3—C4—C5	0.4 (6)	C6—C1—C7—O2	−65.0 (3)
C3—C4—C5—C6	0.3 (6)	C2—C1—C7—O1	−66.0 (3)
C9—O4—C6—C1	174.7 (3)	C6—C1—C7—O1	114.5 (3)

### Hydrogen-bond geometry (Å, °)

**Table:**

*D*—H···*A*	*D*—H	H···*A*	*D*···*A*	*D*—H···*A*
O1—H1···O1^i^	0.77 (4)	1.87 (4)	2.632 (4)	168 (5)
O2—H2···O2^i^	0.79 (5)	1.83 (5)	2.618 (4)	173 (5)

Symmetry codes: (i) −*y*+1, −*x*+1, −*z*+3/2.
